# Small-Molecule Inhibitors and Degraders Targeting KRAS-Driven Cancers

**DOI:** 10.3390/ijms222212142

**Published:** 2021-11-09

**Authors:** Soonsil Hyun, Dongyun Shin

**Affiliations:** 1 College of Pharmacy, Chungbuk National University, 194-21 Osongsaengmyeong 1-ro, Heungdeok-gu, Cheongju-si 28160, Korea; shyun@chungbuk.ac.kr; 2 Gachon Institute of Pharmaceutical Science, College of Pharmacy, Gachon University, 191 Hambakmoe-ro, Yeonsu-gu, Incheon 21936, Korea

**Keywords:** RAS, KRAS mutant, KRAS inhibitors, targeted protein degradation (TPD), drug resistance, PROTAC

## Abstract

Drug resistance continues to be a major problem associated with cancer treatment. One of the primary causes of anticancer drug resistance is the frequently mutated RAS gene. In particular, considerable efforts have been made to treat KRAS-induced cancers by directly and indirectly controlling the activity of KRAS. However, the RAS protein is still one of the most prominent targets for drugs in cancer treatment. Recently, novel targeted protein degradation (TPD) strategies, such as proteolysis-targeting chimeras, have been developed to render “undruggable” targets druggable and overcome drug resistance and mutation problems. In this study, we discuss small-molecule inhibitors, TPD-based small-molecule chemicals for targeting RAS pathway proteins, and their potential applications for treating KRAS-mutant cancers. Novel TPD strategies are expected to serve as promising therapeutic methods for treating tumor patients with KRAS mutations.

## 1. Introduction

*RAS* genes (*HRAS*, *KRAS*, and *NRAS*) are the most frequently mutated genes in cancer cells, showing a mutation frequency of 30% in all cancer cells [1]. Among RAS proteins, KRAS is the predominantly mutated RAS isoform, comprising 85% of oncogenic RAS mutations in cancer cells [2]. *KRAS* mutations causes the most deadliest cancers (lung cancer, colorectal cancer, and pancreatic cancer) [3]. For example, KRAS mutations are seen exclusively in pancreatic ductal adenocarcinoma (PDAC). Alterations in KRAS have been seen in 30% of NSCLC cases, which is the major (80%) form of lung cancer. A few KRAS mutations seen in NSCLC include 39% of G12C, 18–21% of G12V, and 17–18% of G12D [4]. KRAS mutations occur in 35–45% of colon cancers, leading to drug resistance [5].

Despite the advances made in the development of KRAS inhibitors, successful therapies for KRAS mutation-induced cancers, including the direct inhibition of the activity of KRAS and inhibition of downstream KRAS signaling, via the RAF, MEK, and ERK pathways, have not been fully established for several decades [1]. Covalently binding small-molecule inhibitors that bind to new pockets and target KRAS^G12C^ have been identified. These inhibitors have shown potentials in clinical settings, rendering KRAS druggable. In this study, we discuss new strategies for anticancer drug discovery as complementary methods to small-molecule-based strategies. Recently, targeted protein degradation (TPD) has been developed for cancer therapy. A major development in the field of TPD is heterobifunctional small-molecule proteolysis-targeting chimeras (PROTACs), which form a ternary complex between POI and E3 ligase. Thus, PROTAC represents an effective endogenous target protein degradation tool for inducing ubiquitination using the ubiquitin–system (UPS). In this review, we discuss the development of drugs targeting KRAS and KRAS signaling-related proteins, including small-molecule TPD-based chemicals, and their potential applications in treating KRAS mutant cancers.

## 2. Distinct Roles of KRAS in Tumorigenesis and Attempts to Inhibit KRAS Signaling

**Figure 1:**
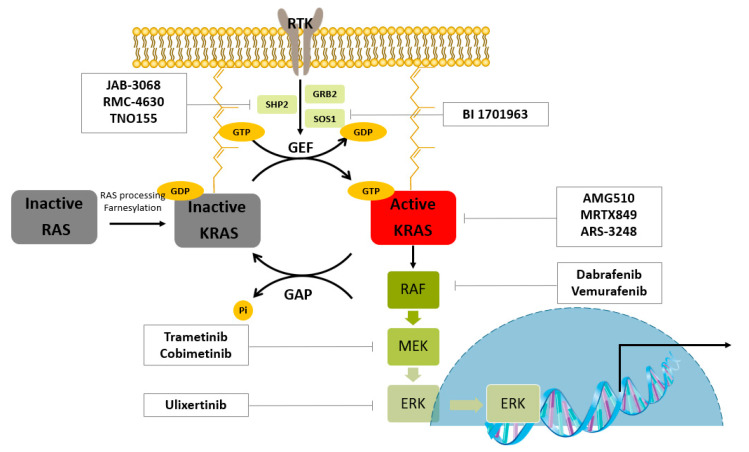
RAS–ERK/MAPK pathway and the representative clinical small molecules for targeted protein degradation. RTK; Receptor tyrosine kinase, GRB2; Growth factor receptor-bound protein 2, SOS1; Son of sevenless homolog 1, SHP2; Src homology-2 domain-containing protein tyrosine phosphatase-2, GEF; guanine nucleotide exchange factor, GAP; GTPase-activating protein, RAF; Rapidly Accelerated Fibrosarcoma, MEK; Mitogen-activated protein kinase, ERK; Extracellular signal-regulated kinase.

KRAS is a prevalent driver of cancer. As shown in Figure 1, the RAS protein is post-translationally modified and localized to the inner plasma membrane. When RAS proteins binds to GTP, they are activated. Guanine nucleotide exchange factors (GEFs) catalyze GDP–GTP exchange processes, and GTPase-activating proteins (GAPs) hydrolyze the nucleotide. One of the GEFs, SOS1, is a RAS activator. The autophosphorylated receptor binds to the growth-factor-receptor-bound protein 2 (GRB2). GRB2 binds to SOS and recruits SOS to the plasma membrane, where RAS is localized. The proximity of SOS to RAS results in increased levels of GTP-bound RAS owing to increased nucleotide exchange. GTP-bound RAS activates effector enzymes and controls cell proliferation. The first mammalian effector protein is RAF. Activated RAF phosphorylates and activates mitogen-activated protein kinase kinases (MEK1 and MEK2). These kinases phosphorylate and activate ERK1 and ERK2. ERK can be transported into the nucleus and phosphorylates transcription factors such as ETS like-1 protein (ELK1) [6].

However, development of inhibitors of this protein has been a challenge for the following reasons: first, selectivity against the RAS family is required to ensure low toxicity; second, the relatively smooth protein structure of the functional GTPase domain of KRAS makes KRAS “undruggable”, which means that designing inhibitors to bind the functional enzyme pocket is difficult [7]; and third, RAS activates downstream signaling of KRAS through protein–protein interactions [8].

### 2.1. Direct Inhibition of RAS

KRAS binds a thousand-fold tighter than ATP; thus, the development of inhibitors that compete with ATP in binding to the kinase domain failed. **SCH-53239** was the first direct inhibitor of KRAS to prevent GDP to GTP conversion, despite toxicity concerns, due to non-selective binding to wtKRAS [9]. To overcome the difficulty of drug development for KRAS, downstream KRAS effectors, such as RAF, MEK, and ERK, also have been targeted.

**Figure 2:**
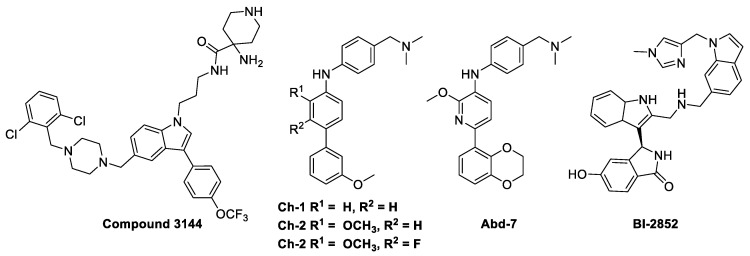
Chemical structures of direct inhibitors of RAS showing the ability to bind to the conserved RAS–effector interaction sites.

**Figure 3:**
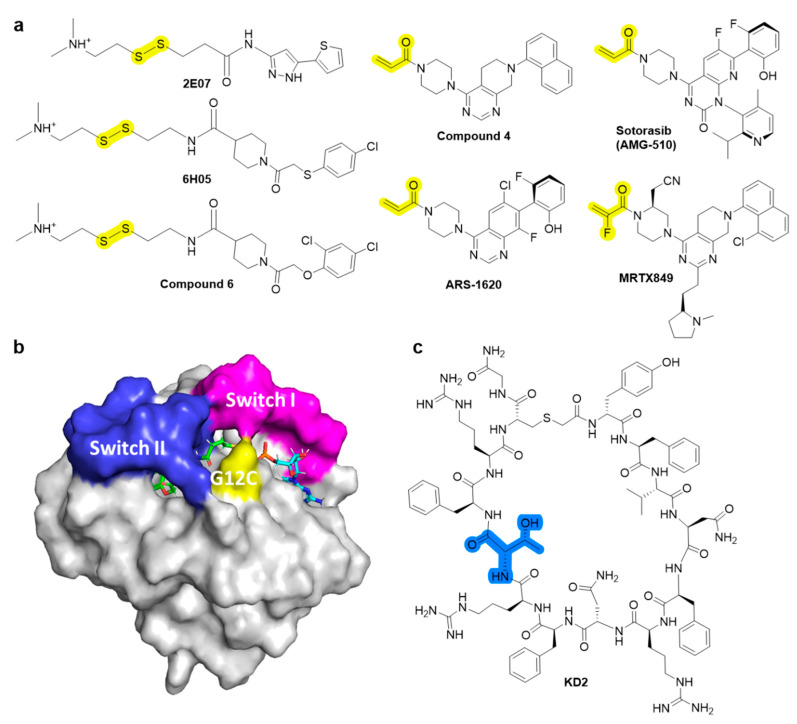
Chemical structures of (**a**) covalent inhibitors of KRAS^G12C^. The functional groups with which Cys at position 12 forms covalent bonds are highlighted in yellow. (**b**) **compound 6** (green) bound in the S-IIP of KRAS^G12C^ with GDP (cyan) (PDB 4LUC). (**c**) A cyclic peptide ligand, **KD2**, of KRAS^G12D^ shown using an integrated in vitro translation–mRNA display selection platform. The Thr residue with which Asp at position 12 of KRAS^G12D^ interacts is highlighted in blue.

Despite intensive attempts to develop anti-RAS small molecules, KRAS was considered “undruggable,” until drugs targeting KRAS mutations were created. Several small molecules [10,11,12,13], which can bind to conserved RAS-effector interaction sites, have been developed to inhibit the RAS family (Figure 2). However, it has been reported that these pan-RAS inhibitors are poorly tolerated owing to their toxicity and off-target effects. Since these pan-RAS inhibitors bind the wild-type RAS proteins and are not selective towards mutant RAS proteins, they need to be further optimized to obtain mutant selectivity. Only the G12C mutation of KRAS has been targeted with effective clinical activities via the formation of a covalent bond between the mutated Cys of KRAS at the G12 position and small molecules. The pioneering work done by Kevan M. Shokat resulted in the finding of the switch-II pocket (S-IIP) [14]. Crystallographic studies reveal that covalent inhibitors bind the new pocket beneath the effector binding switch-II region, thereby inactivating mutant KRAS^G12C^ by preferring GDP to GTP and impairing downstream signaling. Using a disulfide-fragment-based screening approach, a library of 480 compounds was screened for covalent binder to KRAS^G12C^ in the GDP state. Via intact protein mass spectrometry method, the fragments of **2E07** and **6H05** (Figure 3a) showed the greatest modification of the G12C mutant; however, the wild-type KRAS was not modified. Finally, **compound 6** was co-crystalized with the G12C mutant in the GDP state and was found to bind the new allosteric S-IIP, thereby diminishing its interaction with effector proteins such as SOS and RAF. This should act to inhibit the allele-specific KRAS signaling. The identification of the new allosteric pocket provides clues to obtain specificity for the mutant KRAS^G12C^, for which a few covalent inhibitors are currently in clinical trials for. For example, **AMG510** developed by Amgen [15], **MRTX849** developed by Mirati Therapeutics [16], and **ARS-3248** (a derivative of **ARS-1620**) developed by Johnson and Johnson and Wellspring Bioscience [17] were entered into clinical trials for humans in 2018/2019 (Figure 3a) [18]. First, **AMG510** was rationally designed from a lead compound that binds to the allosteric pocket through a custom library synthesis and structure-based design using crystallographic information. A series of compounds with a hybrid scaffold were screened and finally led to the discovery of **AMG 510** with improved drug-like properties suitable for in vivo use [15]. Amgen reported that **AMG 510** led to partial responses in 7 out of 13 patients with NSCLC in Phase I testing. Second, **MRTX849** entered clinical trials in 2019 [16]. Mirati Therapeutics discovered **compound 4** as a lead covalent inhibitor of KRAS^G12C^, screening from a series of tetrahydropyridopyrimidine derivatives. The co-crystal structure of **compound 4** bound to KRAS^G12C^ confirmed the covalent modification of the mutant cysteine trapping KRAS [17]. With modifications of the naphthyl ring and the tetrahydropyridopyrimidine ring, MRTX849 was finally identified to have improved cellular potency and solubility. In preclinical experiments, **MRTX849** inhibited KRAS downstream signaling and tumor growth in patient-derived xenograft models from KRAS^G12C^ mutant tumor cells. In a Phase I/II study, **MRTX849** achieved a partial response in 4 out of 12 patients with NSCLC or colorectal carcinoma, and tumor reduction and shrinking were observed after further treatment with **MRTX849**. Third, Johnson and Johnson and Wellspring Bioscience evaluated **ARS-3248** in clinical trials in the beginning of 2019; however, the trial has been reported to be discontinued [19]. Now, two novel inhibitors, **GDC-6036** and **D-1553**, are currently being evaluated in Phase I trials from Roche and InventisBio, respectively [20].

The development of inhibitors against other KRAS mutations is also important. For example, KRAS^G12D^, which is the most predominant mutation with poor clinical outcomes, is known to impair the intrinsic GTPase activity in G12D mutant [21]. Thus, GTP-bound KRAS^G12D^ is more selectively targeted than wild-type KRAS because of the significant population of GDP bound to wild-type KRAS. Zhang et al. described the discovery of GTP-bound state selective cyclic peptide ligands to KRAS^G12D^ using the Random non-standard Peptides Integrated Discovery (RaPID) platform [22]. Co-crystal structure confirmed that the cyclic peptide ligand, KD2, occupied both the Switch II groove (SIIG) and Switch II pocket (SIIP) through the direct interaction between the Thr residue of **KD2** and the mutant Asp 12. Accordingly, the interactions between KRAS^G12D^ and the Raf1 RAS-binding domain were shown to be inhibited, with an IC_50_ of 12.4 µM, but not in the case of wildtype KRAS at concentrations of up to 100 µM (Figure 3b).

### 2.2. Indirect Inhibition of RAS

#### 2.2.1. Inhibitors of Guanine Nucleotide Exchange Cycle

**Figure 4:**
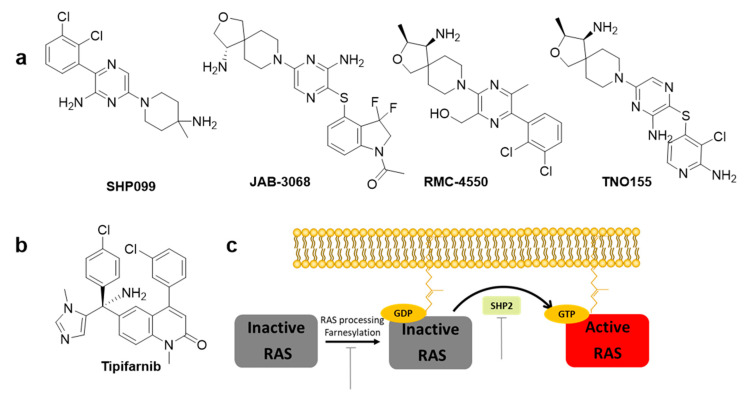
Chemical structures of indirect inhibitors of KRAS. (**a**) SHP2 inhibitors. (**b**) RAS processing inhibitor. (**c**) RAS processing, RAS activation, and target inhibition sites.

Despite the recent successful discovery of clinically applicable inhibitors that targets G12C mutant, targeting strategies for the most prevalent mutants, such as G12D and G12V, still remain unsatisfactory. RAS, a small GTPase, cycles between a GDP-bound inactive state and a GTP-bound active state. RAS mutations accumulate in the GTP-bound state and activate downstream signals, including the mitogen-activated protein kinase (MAPK) and phosphatidylinositol 3-kinase (PI3K) pathways, resulting in tumor growth. Inhibition of GEFs, such as Son of Sevenless homologue 1 (SOS1) or SHP2, reduces the rate of GDP–GTP exchange and the levels of GTP-bound RAS. Thus, inhibitors interfere with positive feedback regulation, which potentiates its GEF function, consequently reducing the survival of tumor cells with KRAS mutation. Currently, there are a few clinically developing GEF inhibitors for monotherapy and are in combination with MEK inhibitors. For example, **BI 1701963** [8], which inhibits the SOS1 and KRAS interactions, and **JAB-3068** [23], **RMC-4630**, and **TNO155** [24,25], which allosterically inhibit SHP2 (Figure 4a).

Using a high-throughput screening of 1.7 million compound libraries, quinazoline ring containing several compounds were identified by Boehringer Ingelheim. Further modifications led to the discovery of **BI-3406**. Co-crystallization studies confirmed a binding pocket next to the catalytic binding site of SOS1 through a pi–pi stacking interaction of His905^SOS^ [1] with the quinazoline ring. **BI-3406** was identified to be a potent nanomolar inhibitor of the protein–protein interaction between SOS1 and KRAS-GDP. Therefore, **BI-3406** reduces cell proliferation and suppresses tumor growth in vivo xenograft models of KRAS-driven cancers. **BI 1701963**, a derivative of **BI-3406**, entered a clinical trial as monotherapy in 2019 and is in combination with the MEK inhibitor trametinib [26].

SHP2 is an attractive target phosphatase that activates downstream RAS signaling of several receptor tyrosine kinases (RTKs); however, its biochemical details have not been elucidated [27]. Traditional SHP2 inhibitors target the protein tyrosine phosphatase binding site and none of the catalytic inhibitors succeeded due to the lack of selectivity over other phosphatases and low bioavailability. However, progress has been made in developing the first selective allosteric inhibitor, **SHP099** (Figure 4a), a structure-based inhibitor with high selectivity. **SHP099** stabilizes the autoinhibited conformation of SHP2 and suppresses RAS-ERK signaling to inhibit the proliferation of human cancer cells in vitro and in mouse patient driven tumor xenograft models [28]. After the identification of the first allosteric inhibitor **SHP099** by Novartis Institutes, several inhibitors, such as **TNO155**, **RMC-4630**, **JAB-3068**, and **JAB-3312**, are under clinical trials. Novartis pharmaceuticals further optimized the pyrazine class of allosteric SHP2 inhibitors and identified **TNO155** as a potent and selective SHP2 allosteric inhibitor [24]. **RMC-4630** developed by Revolution Medicines is currently under clinical Phase I trial as monotherapy (NCT03634982) in combination with the MEK inhibitor cobimetinib (NCT03989115) [29]. **RMC-4630** monotherapy has shown a 71% reduction in the growth of KRAS^G12C^ NSCLC cells in an ongoing Phase 1 clinical trial (NCT03634982) [29]. **JAB-3068** and **JAB-3312** developed by Jacobio are currently under clinical Phase II with AbbVie since May 2020 (NCT04721223 and NCT04720976) [25].

#### 2.2.2. Inhibitors of RAS Processing

RAS proteins are activated when they are localized in the cell membrane. Three post-translational processing enzymes are involved: farnesyltransferase (FTase), geranylgeranyltransferase (GGTase), RAS-converting enzyme (RCE1), and isoprenylcysteine carboxyl methyltransferase (ICMT). Thus, the inhibition of farnesylation blocks cellular membrane insertion of RAS and cancer progression. To inhibit farnesylation of the canonical substrate lamin A, a heterocyclic nonpeptidomimetic drug, tipifarnib (Figure 4b), was developed as the first selective FTase inhibitor with low nanomolar inhibitory concentrations [30]. Tipifarnib is currently under clinical development for treatment of HRAS-mutant thyroid cancer, head and neck squamous cell carcinoma (HNSCC), and non-small-cell lung carcinoma (NSCLC) [31]. Tipifarnib has also been investigated in clinical trials for KRAS mutant NSCLC, but failed since geranylgeranylation can replace prenylation [8,32].

#### 2.2.3. Inhibitors of RAS Effector Proteins

**Figure 5:**
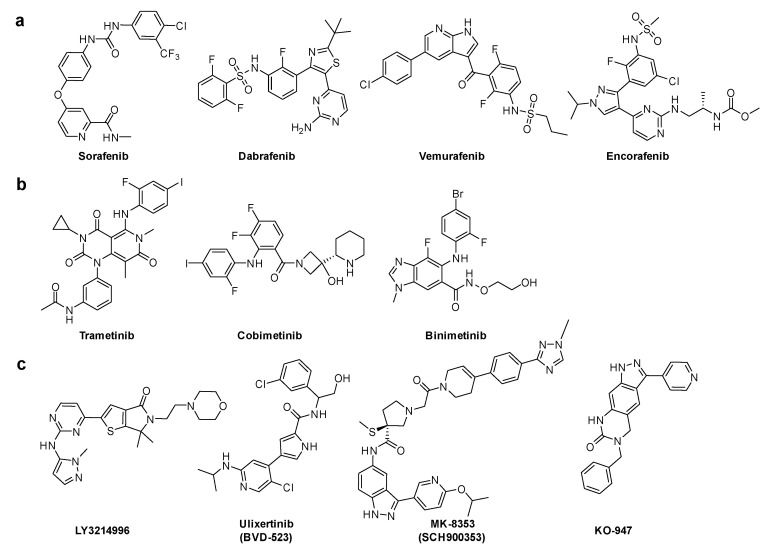
Chemical structures of RAS effector protein inhibitors. (**a**) B-RAF inhibitors. (**b**) MEK inhibitors. (**c**) ERK inhibitors.

Downstream KRAS signaling was investigated through MAPK pathway. GTP-RAS promotes dimerization and phosphorylation of RAF, which activates RAF kinase activity and results in phosphorylation of MEK1 and MEK2. These activated MEK1 and MEK2, and then phosphorylated ERK1 and ERK2. ERK kinases activate growth-transcription factors. First, sorafenib was initially developed to specifically target the ATP binding site of RAF kinase [33]. Although sorafenib is effective in vitro and in vivo in several types of cancer cells, the mode of action of its antitumor activity is later found to have originated from the inhibition of several receptor tyrosine kinases. B-RAF inhibitors, such as dabrafenib, vemurafenib, and encorafenib, are approved antitumor agents against tumors with RAF mutations. These inhibitors are clinically effective in B-RAF^V600E^ melanoma tumors [34]. Second, three MEK inhibitors: trametinib, cobimetinib, and binimetinib have been approved in combination with B-RAF inhibitors. Third, the inhibitors of the final downstream kinase, ERK, are currently in clinical development, including **LY3214496** (NCT02857270) [35], **BVD-523** (NCT03417739) [36], **MK-8353** (NCT01358331) [37], **and KO-947** (NCT03051035) (Figure 5) [1,38].

## 3. Targeted Protein Degraders

### 3.1. Direct KRAS Degrader

**Figure 6:**
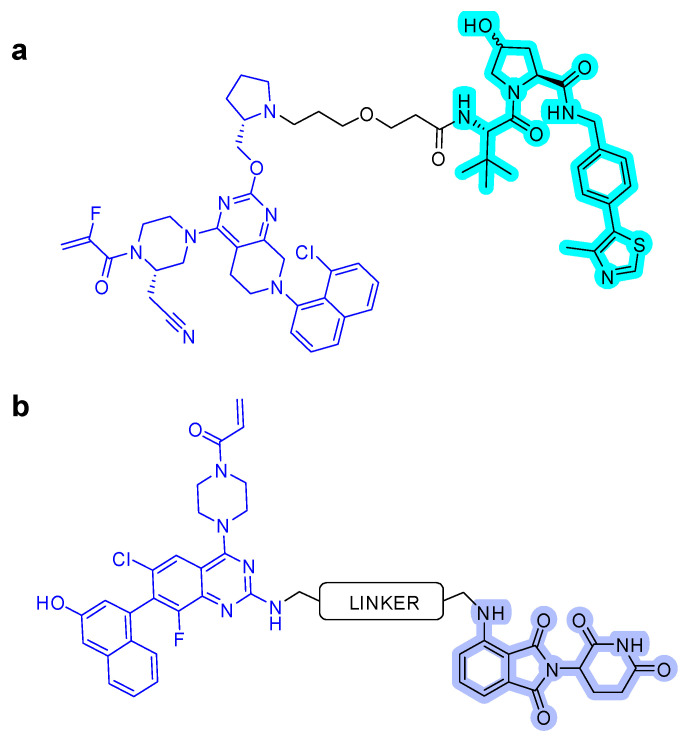
KRAS degrader. (**a**) **LC-2**, MRTX849-VHL PROTAC. (**b**) ARS-1620-CRBN PROTAC. The colors show the part of each compound that it is a structurally important binding motif. The ligands of the POI are in blue. VHL and CRBN ligands are highlighted in cyan and purple, respectively.

After the successful use of a small-molecule inhibitor against KRAS^G12C^, a hetero-bifunctional molecule was explored to test PROTAC-mediated mutant KRAS degradation [39]. The PROTAC targeting KRAS^G12C^, **LC-2**, is composed of the known inhibitor, MRTX849, and the E3 ligase VHL (Figure 6a). The covalent-binding of KRAS^G12C^ with MRTX849 and recruitment of E3 ligase VHL led to successful degradation of KRAS and inhibition of MAPK signaling at submicromolar concentrations [39]. **LC-2** is confirmed to degrade the target protein, KRAS^G12C^, with different efficiencies in both heterozygous and homozygous cells. As expected, 50% degradation of KRAS^G12C^ was observed in heterozygous NCI-H358 cells. In contrast, approximately 75% KRAS^G12C^ degradation was observed in NCI-H2030 cells and MIA PaCa-2 cells each at 0.59 and 0.32 µM of DC_50_ values, respectively. Notably, the degradation specificity of the mutant for wild type protein suggests it as a significant valuable tool for eliminating oncogenic KRAS without interfering with wild-type KRAS function [39].

However, the covalent binding of KRAS^G12C^ with MRTX849 and recruitment of an E3 ligase Cereblon (CRBN) binder, thalidomide derivatives, failed to degrade endogenous KRAS^G12C^ in MIA PaCa-2 and NCI-H358 cells (Figure 6b) [40]. The details of the degradation mechanism of endogenous KRAS^G12C^ are yet to be elucidated; however, heterobifunctional thalidomide conjugated degrader has been shown to successfully recruit CRBN in cells, bind to KRAS^G12C^, dimerize CRBN and KRAS^G12C^, and degrade KRAS^G12C^ in reporter cells.

### 3.2. Degraders of GEFs

The RAS-ERK/MAPK pathway is highly conserved and essential for mammalian development and cellular homeostasis. There are a variety of accessory proteins involved [41]. For example, SHOC2 and SHP2 mediate tumorigenesis in many different cancer cells. Thus, target degradation of accessory regulatory proteins may inhibit not only cancers but also diseases related to the RAS-ERK/MAPK pathways, such as developmental disorders known as RASopathies.

#### 3.2.1. RAS Degrader Using the Protein–Protein Interaction (PPI) between the RAS and Son of Sevenless I (SOS1) Interaction

**Figure 7:**
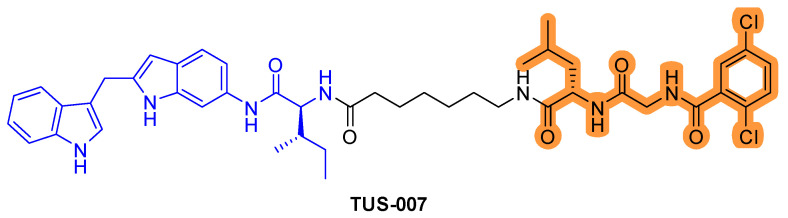
RAS-SOS inhibitor conjugated CANDDY tag. The colors show the part of each compound that it is a structurally important binding motif. CANDDY tag is highlighted in orange.

Although KRAS^G12D^ is commonly observed in pancreatic cancer, there are no effective inhibitors that target KRAS^G12D/V^. Recently, RAS–SOS inhibitor was conjugated with the chemical knockdown with affinity and degradation dynamics (CANDDY) tag (Figure 7) [42]. The CANDDY tag is derived from a proteasome inhibitor, which, unlike current targeted protein degradation techniques, lacks inhibitory activity and induces direct chemical knockdown without ubiquitination. The CANDDY tag conjugated RAS–SOS1 inhibitor TUS-007 suppresses tumors by degrading KRAS^G12D/V^ in a dose-dependent manner and suppresses in vivo tumor growth [42].

#### 3.2.2. SHP2 Protein Degrader

**Figure 8:**
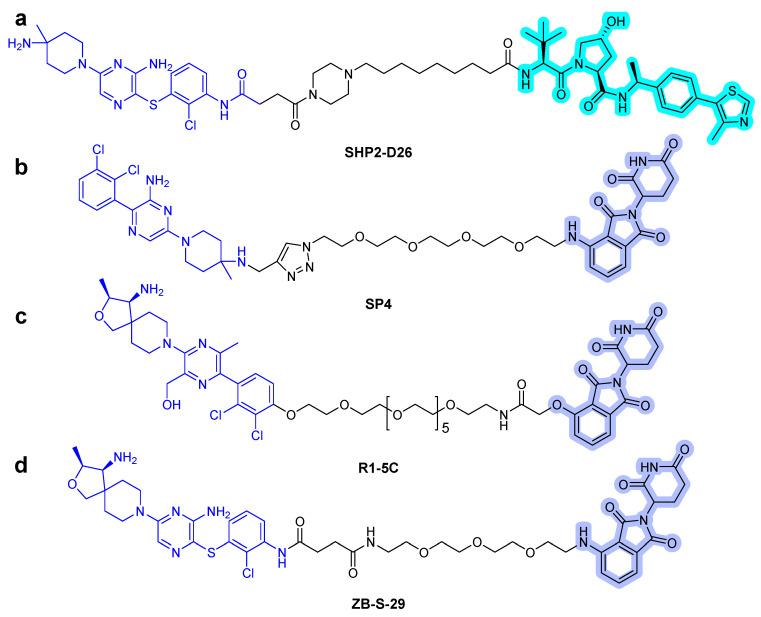
SHP2 degraders. (**a**) SHP099-derived SHP2 binder-VHL PROTAC. (**b**) SHP099-CRBN PROTAC. (**c**) RMC-4550-CRBN PROTAC. (**d**) TNO155-CRBN PROTAC. The colors show the part of each compound that is a structurally important binding motif. The ligands of the POI are depicted in blue color. VHL and CRBN ligands are highlighted in cyan and purple colors, respectively.

Recently, Wang et al. described the discovery of a potent SHP2 degrader. The designed heterobifunctional molecules consisted of SHP099-derived SHP2 binder, VHL ligand, and alkyl/PEG-based linkers [43]. They successfully degraded the target protein, and among them, **SHP2-D26** (Figure 8a) presented high degradation potency with 2.6 and 6.0 nM of DC_50_ (50% degradation concentration) in acute myeloid leukemia MV4-11 and esophageal cancer KYSE520 cells, respectively. Moreover, it eliminated the target protein by over 95% in cancer cells. Compared with the small-molecule inhibitor SHP099, the PROTAC compound **SHP2-D26** was shown to inhibit the phosphorylation of ERK and cell growth by more than 30-fold.

The conjugation of CRBN ligands with the ligands of the protein of interest (POI) is another approach to generate heterobifunctional molecules [44]. In 2021, three articles, which described thalidomide-based SHP2 PROTAC compounds, were published [44]. Zheng et al. designed SHP2 degraders [45]. They utilized SHP099 as the SHP2 binder and determined the terminal amine group as the position of the linking vector and synthesized the SP series that linked POM and SHP099 with PEG. Eventually, the effective **SP4** PROTAC (Figure 8b) was found. Its inhibitory activity against the growth of HeLa cells was 100 times greater than that of SHP099. Vemulapalli et al. designed a novel SHP2 PROTAC, generated by connecting pomalidomide and RMC compound, using PEG linkers [46]. The synthesized SHP2 PROTAC **R1-5C** (Figure 8c) selectively degraded SHP2 protein in MV4-11 and MOLT cells and successfully inhibited cell growth. Yang et al. reported SHP2 degraders, and an analog of TNO155 (IC_50_ = 11 nM) [24] was chosen as a potent SHP2 binder and conjugated with thalidomide [44]. One of the heterobifunctional molecules, **ZB-S-29** (Figure 8d), has been identified to be more effective in inhibiting cell growth of MV4-11 cells than SHP099 via effective degradation of SHP2 protein, with a DC_50_ of 6.02 nM. The conjugation of RMC-4550 [47], an SHP2 allosteric inhibitor, with POM induced degradation of SHP2 in leukemia cell lines, such as KYSE520 and MV4-11 cells, with a low nanomolar DC_50_.

All these SHP2 degraders provide alternative approaches to the inhibition of SHP2-mediated RAS signaling pathways other than conventional inhibitors. These SHP2 degraders provide a useful tool for the acute depletion of SHP2 in functional studies.

### 3.3. RAF Degrader

**Figure 9:**
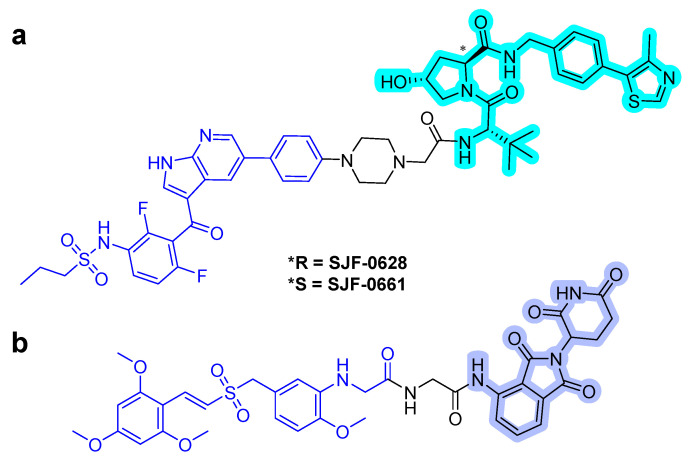
BRAF degrader. (**a**) Vemurafenib-based PROTAC. (**b**) Rigosertib-CRBN PROTAC. A stereocenter is indicated by an asterisk. The colors show the part of each compound that is a structurally important binding motif. The ligands of the POI are in blue. VHL and CRBN ligands are highlighted in cyan and purple, respectively.

Activated RAF phosphorylates MEK activates ERK to promote cell proliferation A potent oncogenic B-RAF mutations, which occur in 8% of all cancers [48]. FDA-approved B-RAF inhibitors, such as vemurafenib, have been successfully developed for patients with the B-RAF V600E mutation. Using the conjugates of E3 ligase ligand (e.g., VHL) with vemurafenib, BRAF mutants were shown to be efficiently degraded at low nanomolar concentrations without degrading wtBRAF (Figure 9a) [49]. Rigosertib, a non-ATP-competitive inhibitor of PLK1, was also developed as PROTAC via conjugation with pomalidomide as a BRAF degrader (Figure 9b) [50].

### 3.4. MEK1/2 Degrader

Mitogen-activated protein kinases 1 and 2 (MEK1/2) are one of the crucial enzymes of the ERK pathway [51]. The use of MEK inhibitors is limited due to the acquired resistance towards them in patients undergoing long-term treatment. However, since mutations in MEK1/2 induce resistance to MEK inhibitors, the opportunity to overcome resistance should be investigated via MEK1/2 degradation and PROTAC technology [52,53,54].

**Figure 10:**
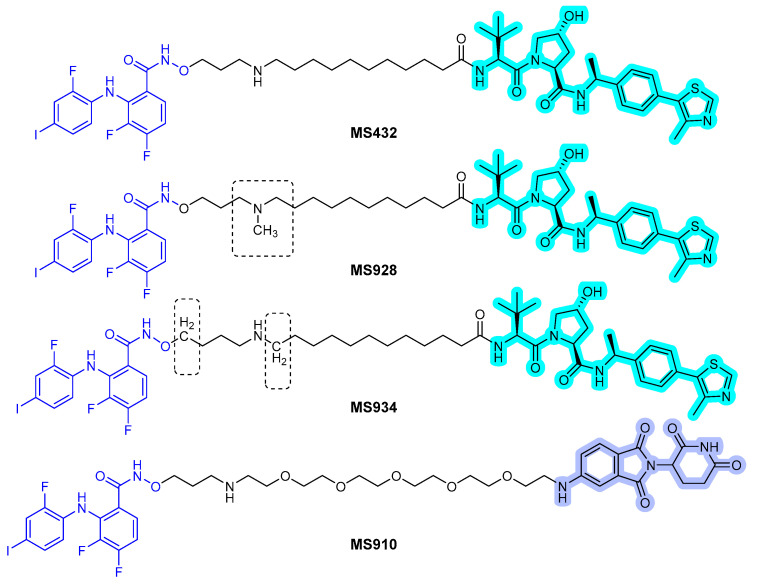
MEK1/2 degraders. The dashed boxes in MS928 and MS934 indicate the part of each compound that differs in structure from MS432. The colors show the part of each compound that is a structurally important binding motif. The ligands of the POI are in blue. VHL and CRBN ligands are highlighted in cyan and purple, respectively.

In 2019, Wei et al. reported a first-in-class MEK1/2 degrader [52]. They used polyhalogenated diphenylamines, which were designed based on the structures of the known MEK1/2 inhibitors: cobimetinib and binimetinib as MEK1/2 binder. Both VHL and CRBN binders were applied to the MEK1/2 PROTACs, and simple alkyl and PEG lingers were used. Degradation assay revealed that the PROTAC **MS432** (Figure 10) with the VHL binder and C10 alkyl linker exhibited potent degradation activities with pharmacological profiles of 31 ± 9 nM of DC_50_ for MEK1, 17 ± 2 nM of DC_50_ for MEK2, and 130 ± 38 nM of GI_50_ in HT29 cells and 31 ± 1 nM of DC_50_ for MEK1, 9.3 ± 5 nM of DC_50_ for MEK2, and 83 ± 15 nM of GI_50_ in SK-MEL-28 cells. Moreover, the PROTAC compound was shown to be highly MEK1/2 selective with regards to global proteomic profiling and favorable bioavailability.

In 2020, the same group reported the result of an extensive structure-activity relationship study of **MS432** [53]. They optimized the types and length of the linkers to obtain two improved MEK1/2 PROTACs, **MS928** and **MS934** (Figure 10), by maintaining the MEK1/2 binder and VHL E3 ligase binder. **MS934** presented pharmacological profiles of 18 ± 1 nM of DC_50_ for MEK1, 9 ± 3 nM of DC_50_ for MEK2, and 23 ± 5 nM of GI_50_ in HT29 cells and 10 ± 1 nM of DC_50_ for MEK1, 4 ± 1 nM of DC_50_ for MEK2, and 40 ± 10 nM of GI_50_ in SK-MEL-28 cells. In addition, the first MEK1/2 degrader (**MS910**) with a CRBN E3 ligase binder was found.

**Figure 11:**
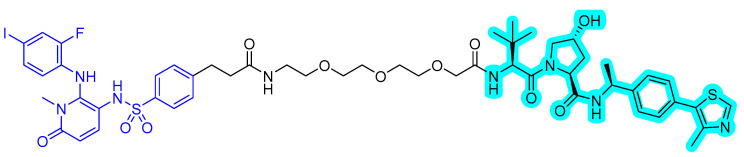
MEK1/2 degrader. The colors show the part of each compound that is a structurally important binding motif. The ligand of the POI is in blue. VHL ligand is highlighted in cyan.

In 2020, a new series of MEK PROTACs was reported by Voller et al. [54]. Until now, two allosteric MEK inhibitors, trametinib and cobimetinib, have been approved. The research group designed the MEK binder using the crystal structure of MEK with refametinib (PDB 3E8N). The vector for linker attachment was determined through analysis of the binding mode of the inhibitor. Several VHL-recruiting MEK PROTACs were synthesized and screened for degradation to obtain an effective MEK PROTAC, **compound 3** (Figure 11), which degraded MEK1 up to 91% at 1 μΜ after 16 h.

**Figure 12:**
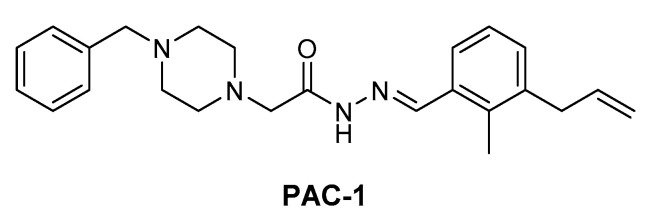
Procaspase activating compound 1 (**PAC-1**).

MEK1/2 kinases were revealed to be degraded by caspase-3 during apoptosis [55,56]. In 2018, Peh et al. reported novel indirect MEK1/2 degrader. The discovered procaspase activating compound has piperazinyl acylhydrazone scaffold that is named PAC-1 (Figure 12), which structurally is not hetero-bifunctional PROTAC molecule. PAC-1 has shown to directly activate procaspase-3 to caspase-3 and consequently induce MEK1/2 degradation [57]. In this article, the authors showed the combination effect of PAC-1 with vemurafenib, osimertinib, and ceritinib against BRAF^V600E^ melanoma, EGFR^T790M^ lung cancer, and EML4-ALK lung cancer and in all cancer cell-lines, and the acquisition of resistance was substantially delayed or eliminated.

However, as PAC-1 targets multiple proteins such as DNA polymerase and some transcription factors, it is likely that PAC-1 may have off-target effects. Therefore, PROTAC strategy for degrading MEK1/2 might have significant advantages over PAC-1 in terms of applications and low toxicities.

### 3.5. ERK Degraders

PROTACs are hetero-bifunctional molecules that consist of three components: POI binding motif, E3 ligase binding motif, and linking motif. Consequently, the molecular weights of PROTAC molecules are much higher than that of common small-molecule drugs. In the course of optimizing lead compounds to drug candidates, the characteristic of PROTACs can limits drug-like properties such as solubility, lipophilicity, membrane permeability, and pharmacokinetic parameters. To overcome the inherent drawbacks of PROTACs, Lebraud et al. investigated a variant technology of PROTAC. They envisioned in-cell assembly of POI binding motif and E3 ligase binding motif through bio-orthogonal click chemistry to generate in situ PROTAC molecules, named in-cell click-formed proteolysis-targeting chimeras (CLIPTACs) [58]. They proved the technological concept by designing a trans-cyclo-octene (TCO) tagged POI binder, which react with tetrazine tagged E3 ligase binders in cells to form CRBN recruiting PROTACs. Through CLIPTAC, several POIs were successfully degraded.

**Figure 13:**
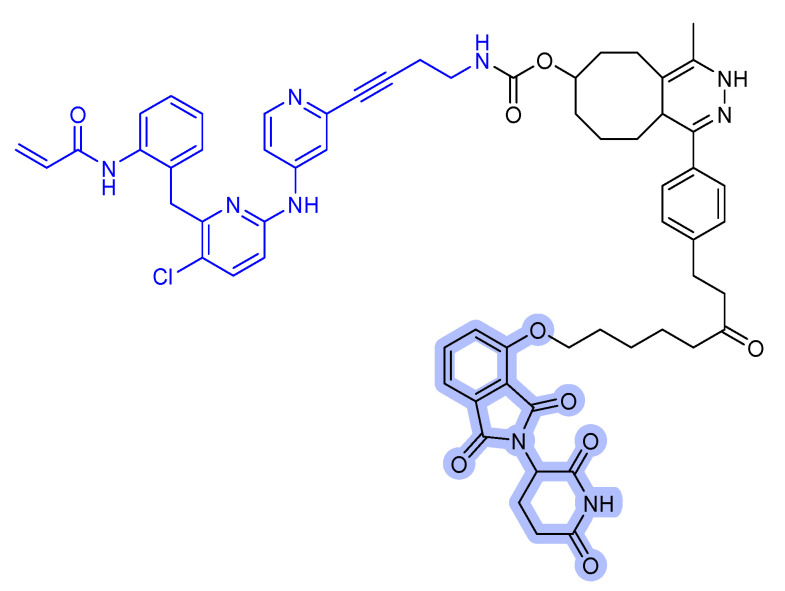
ERK-CLIPTAC. The colors show the part of each compound that it is a structurally important binding motif. The ligand of the POI is in blue. CRBN ligand is highlighted in purple.

The first ERK1/2 degrader, named ERK-CLIPTAC, was synthesized by an in cell CLICK reaction between ERK1/2 covalent inhibitor (TCO Probe 1) and a tetrazine-tagged CRBN ligand (Figure 13) [58]. By the in-cell click-formation of a heterobifunctional molecule, ERK1/2 degradation was achieved at low micromolar concentration. Notably, ERK-CLIPTAC is the first example showing the usage of covalent warhead, and therefore, it is expected to require a stoichiometric amount for theoretically complete target protein degradation. ERK1/2 degradation was achieved at low micromolar concentrations by the in-cell click formation of a heterobifunctional molecule.

## 4. Perspectives and Conclusions

Resistant cancers caused by KRAS mutations are a significant problem in current clinical cancer treatments. TPD is a novel strategy for drug-resistant targets. Conventional occupancy-driven inhibitors inhibit protein function by binding only to protein active sites. However, these inhibitors induce changes in the expression levels of other related proteins via feedback mechanisms. Therefore, the novel catalytic degradation strategy of target proteins can enable the inhibition of all protein functions and may be a complementary strategy, along with small-molecule inhibitors, for combatting drug resistance in tumors. In addition, the PROTAC approach may provide additional opportunities to develop drugs that target “undruggable” proteins by using small molecules that bind to allosteric or even non-functional binding sites. As shown in previous clinical trials, resistance has been overcome by PROTACs, targeting AR, ER, BTK, BET, and BCR-ABL [59,60,61]. Therefore, a novel TPD strategy is a promising strategy for tumor patients with KRAS mutations using direct degradation approaches of KRAS, accessory proteins, such as SHP2 and SOS1, and downstream effective proteins.

## Data Availability

Not applicable.
